# Characterization of fusion genes in common and rare epithelial ovarian cancer histologic subtypes

**DOI:** 10.18632/oncotarget.16781

**Published:** 2017-04-01

**Authors:** Madalene A Earp, Rama Raghavan, Qian Li, Junqiang Dai, Stacey J. Winham, Julie M. Cunningham, Yanina Natanzon, Kimberly R. Kalli, Xiaonan Hou, S. John Weroha, Paul Haluska, Kate Lawrenson, Simon A. Gayther, Chen Wang, Ellen L. Goode, Brooke L. Fridley

**Affiliations:** ^1^ Department of Health Sciences Research, Mayo Clinic, Rochester, MN, USA; ^2^ Department of Biostatistics, University of Kansas Medical Center, KS, USA; ^3^ Department of Laboratory Medicine and Pathology, Mayo Clinic, Rochester, MN, USA; ^4^ Division of Medical Oncology, Mayo Clinic, Rochester, MN, USA; ^5^ Department of Oncology, Mayo Clinic, Rochester, MN, USA; ^6^ Department of Molecular Pharmacology and Experimental Therapeutics, Mayo Clinic, Rochester, MN, USA; ^7^ Women's Cancer Program at the Samuel Oschin Comprehensive Cancer Institute, Cedars-Sinai Medical Center, Los Angeles, CA, USA; ^8^ Center for Cancer Prevention and Translational Genomics, Samuel Oschin Comprehensive Cancer Institute, Cedars-Sinai Medical Center, Los Angeles, CA, USA; ^9^ Department of Biomedical Sciences, Cedars-Sinai Medical Center, Los Angeles, CA, USA; ^10^ Department of Biostatistics and Bioinformatics, Moffitt Cancer Center, Tampa, FL, USA

**Keywords:** fusion gene, ovarian cancer, RNA-sequencing, histological subtypes, TCGA

## Abstract

Gene fusions play a critical role in some cancers and can serve as important clinical targets. In epithelial ovarian cancer (EOC), the contribution of fusions, especially by histological type, is unclear. We therefore screened for recurrent fusions in a histologically diverse panel of 220 EOCs using RNA sequencing. The Pipeline for RNA-Sequencing Data Analysis (PRADA) was used to identify fusions and allow for comparison with The Cancer Genome Atlas (TCGA) tumors. Associations between fusions and clinical prognosis were evaluated using Cox proportional hazards regression models. Nine recurrent fusions, defined as occurring in two or more tumors, were observed. *CRHR1-KANSL1* was the most frequently identified fusion, identified in 6 tumors (2.7% of all tumors). This fusion was not associated with survival; other recurrent fusions were too rare to warrant survival analyses. One recurrent in-frame fusion, *UBAP1-TGM7*, was unique to clear cell (CC) EOC tumors (in 10%, or 2 of 20 CC tumors). We found some evidence that CC tumors harbor more fusions on average than any other EOC histological type, including high-grade serous (HGS) tumors. CC tumors harbored a mean of 7.4 fusions (standard deviation [sd] = 7.4, *N* = 20), compared to HGS EOC tumors mean of 2.0 fusions (sd = 3.3, *N* = 141). Few fusion genes were detected in endometrioid tumors (mean = 0.24, sd = 0.74, *N* = 55) or mucinous tumors (mean = 0.25, sd = 0.5, *N* = 4) tumors. To conclude, we identify one fusion at 10% frequency in the CC EOC subtype, but find little evidence for common (> 5% frequency) recurrent fusion genes in EOC overall, or in HGS subtype-specific EOC tumors.

## INTRODUCTION

A fusion gene, or chimera, is a hybrid gene formed from the aberrant juxtaposition of two distinct genes. Events leading to a fusion gene can occur at the DNA level through translocation, deletion, or inversion, or at the RNA level as a result of read-through transcription or trans-splicing (when a single RNA transcript is processed from multiple separate pre-mRNAs) [1, 2]. Gene fusions play a critical role in some cancers, either by altering expression levels, or functionality, or both [1]. A well-described example is *BCR-ABL1*, a fusion gene that confers tumor growth factor independence, inhibits apoptosis, and is the defining molecular aberration in chronic myelogenous leukemia (CML) (95% of cases) [3]. *BCR-ABL1* is the target for the highly selective drug imatinib (Gleevec^®^), whose development is largely responsible for nearly doubling the 5-year survival time of CML patients [1, 4]. In solid tumors, recurrent gene fusions have been described in prostate cancer (*TMPRSS2-ERG*), lung cancer (*EML4-ALK*), and secretory breast cancer (*ETV6-NTRK3*) [5–7]. *TMPRSS2-ERG* has been reported in >50% of tumors, and has been used to stratify patients according to risk [8] and survival time [9]. These examples demonstrate that recurrent gene fusions have the potential for clinical benefit as drug targets and may have diagnostic and prognostic uses.

Epithelial ovarian cancer (EOC) affects 1.3% of women and has poor 5-year survival (~46%) in comparison to other women's cancers such as breast (~90%), endometrial (~83%), and cervical (68%) [4]. This is in part due to a lack of effective early detection strategies, and a high recurrence rate with only modest activity from standard second-line chemotherapies [10]. Recent advances in the understanding of EOC include the appreciation of histologically and molecularly defined subtypes. With regard to these molecular features, relatively few studies have investigated recurrent fusions in a large panel of EOC tumors by histological subtype. High-grade serous (HGS) EOC, the most common and lethal subtype, has been investigated for fusion genes as part of The Cancer Genome Atlas (TCGA) project [11, 12]. Among 400 tumors studied, very few recurrent fusions were detected, and nearly all of those that were found were in genomics regions with copy number alteration [12]. This led the authors conclude that fusions in HGS EOC arise secondary to the widespread genomic instability characteristic of this subtype [12]. The recurrent fusions reported in TCGA's EOC tumors were *CCDC6-ANK3* (in 4 samples, 1% of tumors), and *COL14A1-DEPTOR* and *KAT6B-ADK* (each in 2 samples, 0.5% of tumors). Another recent molecular profiling study of 114 HGS EOC tumors found a similar low frequency of recurrent fusion genes. The only recurrent fusion reported was *SLC25A40-ABCB1*, detected in two chemotherapy-resistant relapsed tumor samples [13]. Overall, the low frequency of recurrent fusions in HGS EOC limits their potential clinical use.

The role that fusion genes play in the rarer EOC histological subtypes, including endometrioid (END), clear cell (CC), and mucinous (MC) tumors is less clear. Previous studies were limited by small sample size (≤ 8 END, ≤ 16 CC, and ≤ 2 MC) [14–17]. Thus, we screened for recurrent gene fusions using paired-end (PE) RNA sequencing (RNA-seq) of a histologically diverse panel of well characterized EOCs (141 HGS, 55 END, 20 CC, and 4 MC) with clinical outcome data.

## RESULTS

### Fusion genes by EOC subtype

**Figure 1:**
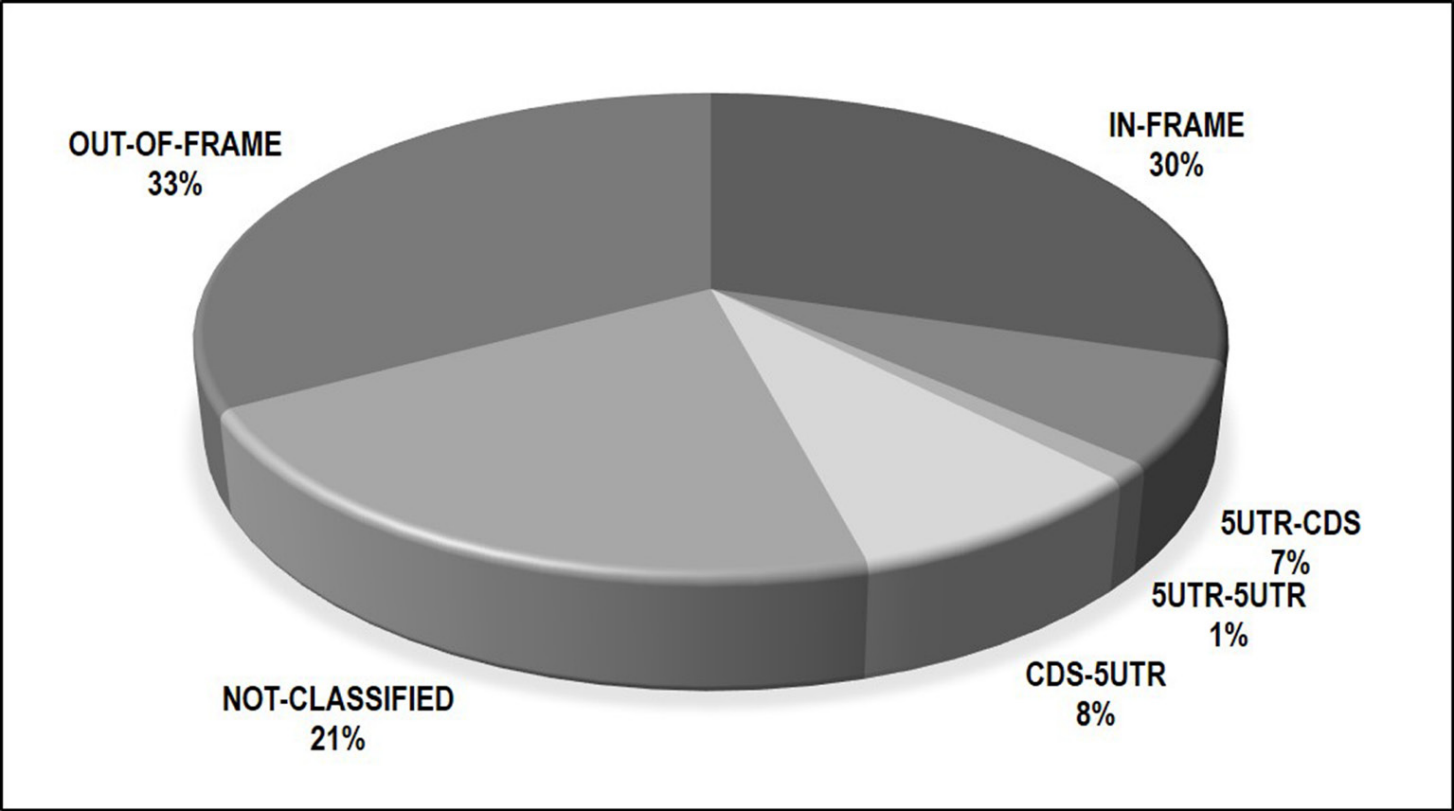
Breakdown of 442 fusions by predicted consequence to reading frame

Following the application of strict filtering criteria, we identified 442 unique fusion genes across 220 EOC tumors. In a given tumor, fusions with multiple predicted breakpoints involving the same gene partners were considered a single event. These 442 fusions were categorized by predicted functional type, shown in Figure 1. These predictions were made by PRADA based on the position of the fusion junction sites (given in human genome build 37). Approximately one third of fusions (30%, *N* = 134) are predicted to be in-frame, meaning the involved gene partners have a preserved reading frame, and are potentially functional if translated. Another 16% (*N* = 71) involved exchanges of 5′-untranslated region (UTR). UTR's contain regulatory elements that can alter expression levels of genes they are positioned next to. Finally, 33% of fusions were out of frame and likely degraded; 21% were not-classified by PRADA. All fusions and their annotations are given in Supplementary Table 1.

**Figure 2:**
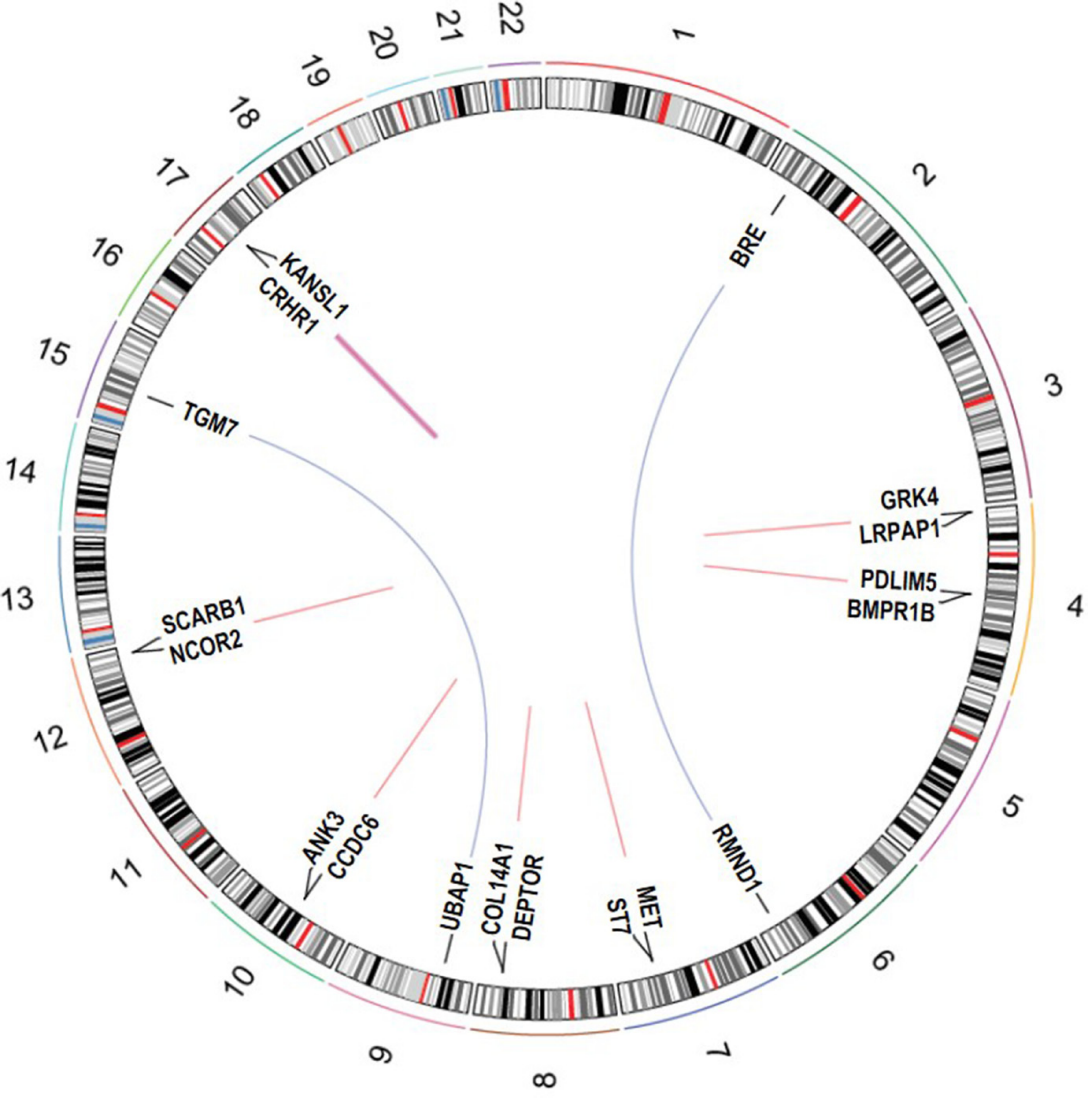
Circos plot of recurrent fusion genes detected in 220 EOC tumors The outer circle shows cytogenetic bands (based on Circos package data UCSC.hg19.chr).

Fusion genes detected in two or more tumor, i.e. recurrent fusions, are summarized in Table 1 and Figure 2. Two fusions were inter-chromosomal, involving gene partners on different chromosomes (*RMND1-BRE* and *UBAP1-TGM7*); the remaining seven involved genes in close proximity (< 1 Mb) on the same chromosome (intra-chromosomal). The fusion *UBAP1-TGM7*, detected in two CC tumors, was also validated using Sanger Sequencing (Supplementary Figure 1). Fusions involving genes less than 1Mb away on the same strand are likely to reflect read-through transcripts [18]. Five fusions in Table 1 fit this criterion. Many of the fusions in Table 1 are predicted to be in-frame, including both of the inter-chromosomal fusions. The most recurrent fusion was *CRHR1-KANSL1* in 6 tumors (2.7% of all samples); all other recurrent fusions were found in two tumors (1% of all samples). *UBAP1-TGM7* was unique to CC tumors (in 10%, or 2 of 20, CC tumors). One recurrent fusion, *ST7-MET*, was found in 1 HGS and 1 CC tumor. All other recurrent fusions were found in HGS tumors only. No recurrent fusions were unique to relapsed or post-NACT tumors, albeit our sample size for tumors with these clinical characteristics was small (6 for each).

**Table 1:** Recurrent fusions detected in epithelial ovarian cancer tumors

Fusion Type^1^	Recurrent Fusion (Gene A-Gene B)^2^	No. of Tumors	Tumor Histology	Strand (Gene A/ Gene B)	Consequence to reading frame^3^	Likely to be expressed and functional^4^	Found by SoapFuse^5^	Reported in OV-TCGA/any TCGA^6^
Interchromosomal	RMND1-BRE	2	2 High-Grade Serous*	−/+	In-frame	Yes	Yes	No/No
	UBAP1-TGM7	2	2 Clear Cell	+/−	In-frame	Yes	No	No/No
Intrachromosomal	CRHR1-KANSL1	6	4 High-Grade Serous 2 Clear Cell	+/−	Not classified	Unknown	No	No/Yes
	LRPAP1-GRK4	2	2 High-Grade Serous#	−/+	In-frame	Yes	Yes	No/No
Intrachromosomal, Read-through	CCDC6-ANK3	2	2 High-Grade Serous	−/−	In-frame/not-classified	Yes/ Unknown	Yes	Yes/Yes
	NCOR2-SCARB1	2	2 High-Grade Serous	−/−	In-frame/out-of-frame	Yes, No	Yes	No/Yes
	ST7-MET	2	1 High-Grade Serous#	+/+	In-frame/out-of-frame	Yes, No	No	No/Yes
			1 Clear Cell					
	BMPR1B-PDLIM5	2	2 High-Grade Serous	+/+	5-UTR to CDS/not-classified	Unknown	Yes	No/No
	COL14A1-DEPTOR	2	2 High-Grade Serous	+/+	Out-of-frame	No	No	Yes/Yes

* One tumor exposed to neoadjuvant chemotherapy. #One relapsed tumor. Abbreviations used: UTR, untranslated region; CDS, coding DNA sequence; OV, ovarian; TCGA, The Cancer Genome Atlas. TCGA fusion gene data available at: http://54.84.12.177/PanCanFusV2/.

^1^Interchromosomal fusions involve gene partners on different chromosomes. Intrachromosomal fusions involve gene partners on the same chromosome. Read-through fusions involve gene partners less than 1 Mb away on the same strand.

^2^Gene A is 5′ gene; Gene B is 3′ gene.

^3^As predicted by the fusion caller PRADA based on fusion junction sites (in GRCh37/hg19, see Supplementary Table 1 for positions).

^4^“Yes” if predicted protein reading frame is in-frame, “No” if reading frame is Out-of-Frame, “Unknown” for all other cases.

^5^“Yes” if fusion was also detected by the fusion caller SoapFuse; “No” if it was not.

^6^“Yes” if fusion was also reported in OV-TCGA tumors or any organ site TCGA tumors; “No” if it was not.

Studies have underscored limitations associated with relying on any one fusion caller to detect fusions [19–21], therefore, we further examined whether the fusions in Table 1 were also detected by the robust fusion caller SoapFuse [22]. Five recurrent fusions detected by PRADA were also detected by SoapFuse (see Table 1). Two of the nine recurrent fusions identified, *CCDC6-ANK3* and *COL14A1-DEPTOR*, have previously been reported in TCGA's EOC tumors. In our tumor set, the histology-specific frequency of these fusion genes was 1.4% (2 of 141 HGS samples). In TCGA, their frequency was low at 1% (*CCDC6-ANK3*) and 0.5% (*COL14A1-DEPTOR*). Examining all tumor types in TCGA, two additional fusions in Table 1 are noted as being previously been reported. *CRHR1-KANSL1* fusions were reported in 5% of TCGA's colorectal tumors (16 of 312 analyzed), and 3.5% of TCGA's uterine carcinosarcomas (2 of 57 analyzed) [12]. *NCOR2-SCARB1* fusions were found in five different TCGA tumor types at a frequency < 2%. In addition to being found in EOC tumors, *CCDC6-ANK3* fusions were reported at low frequencies in breast (2 of 1140) and lung (1 of 546) tumors. A *COL14A1-DEPTOR* fusion was also found in 1 lung tumor in TCGA.

**Figure 3:**
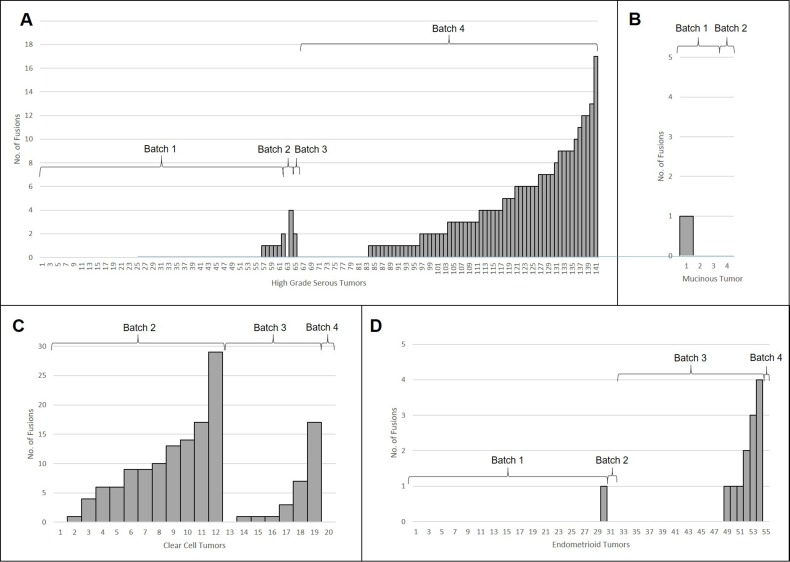
Histology-specific histograms showing how the number of fusions detected per tumor in: (**A**) High Grade Serous, (**B**) Mucinous, (**C**) Clear Cell, and (**D**) Endometrioid. RNA-seq batch is also indicated.

The EOC tumors included in our study harbored a mean of 2 and a median of 0 fusions; 129 (59%) tumors had no fusions detected. The average number of fusions detected in tumors differed by histology (p_ANOVA_ = 5.3 × 10^−12^) (Table 2, Figure 3). Pairwise comparison between the histologies, using *t*-tests assuming unequal variance with Bonferroni adjustment for multiple comparisons, found significant differences in number of fusions detected between CC and the three other histologies (p_HGS-CC_ = 0.033, p_END-CC_ = 0.003, p_MC-CC_ = 0.003); additional there was significance differences between HGS and the remaining two histologies (p_HGS-END_ = 3.9 × 10^−8^, p_HGS-MC_ =0.002). CC tumors had the highest average number of fusions (mean = 7.4, sd = 7.6), followed by HGS tumors (mean = 2.0, sd = 3.3), then END (mean = 0.24, sd = 0.74) and MC (mean = 0.25, sd = 0.5) tumors. It should be noted, however, that these results could be attributed to the batch issues, for which we are unable to untangle this confounding. Therefore, we also looked for histologies differences within given batches of samples. The average number of fusions was compared across histology within RNA-seq batch to further examine histology-specific differences. CC tumors harbored more fusions on average than the other EOC subtypes in RNA-seq Batch 2 (*p* = 0.002, *t*-test assuming unequal variance, 12 CC compared to 5 non-CC subtypes), but not Batch 3 (*p* = 0.16, *t*-test assuming unequal variance, 7 CC compared to 23 non-CC subtypes). No CC tumors were sequenced in Batch 1 and only 1 CC tumor was in Batch 4 (no fusions were detected in this tumor) (Table 1). No other statistically significant histology specific differences in the number of fusions per tumor were observed.

**Table 2:** Average No. of fusions detected in tumors by batch and histology

Histology	Batch				Histology Average
	1	2	3	4	
High Grade Serous	0.11 (62)	2.00 (2)	2.00 (2)	3.59 (76)	2.03 (141)
Endometrioid	0.00 (30)	0.00 (2)	0.55 (22)	0.00 (1)	0.24 (55)
Clear Cell	NA (0)	9.83 (12)	4.29 (7)	0.00 (1)	7.40 (20)
Mucinous	0.33 (3)	0.00 (1)	NA (0)	NA (0)	0.25 (4)
**Batch Average**	0.09 (95)	7.18 (17)	1.47 (30)	1.47 (78)	2.0 (220)

* Bracketed values are the number of tumors in a category. ‘NA’ indicates a category did not contain any tumors.

### Prognostic significance and clinical features of fusion genes by EOC subtype

Fusion gene status, i.e. the presence or absence of any fusion gene, was not significantly associated with overall survival (OS) time (*p* = 0.34) or progression free survival (PFS) time (*p* = 0.06). Adjustment for histology and RNA-seq batch did not change this conclusion (OS *p* = 0.82, PFS *p* = 0.49). *CRHR1-KANSL1*, detected in 6 tumors, was not associated with OS (*p* = 0.59) or PFS (*p* = 0.85); other recurrent fusions were too rare to warrant survival analyses. We also grouped our detected fusion genes into 4 classes, based on established functional relevance in tumorigenesis [12]: kinases (*N* = 21 fusions, gene list from [12]); tumor suppressor genes (TSG) (*N* = 9 fusions, gene list from https://bioinfo.uth.edu/TSGene/); chromatin modifiers (*N* = 13 fusions); and histone modifiers (*N* = 25 fusions). The latter two genes lists were from http://epifactors.autosome.ru/. None of these four protein classes were associated with outcome. Tumors fusion gene status was significantly associated with presence of endometriosis (*p* = 0.008); however other clinical or lifestyle features were not associated fusion status, after adjusting for histology and batch (i.e., grade (*p* = 0.71), presence of ascites (*p* = 0.35), peritoneal cytology (*p* = 0.83), debulking status (*p* = 0.28), smoking history (*p* = 0.49), age at first live birth (*p* = 0.58)).

## DISCUSSION

**Figure 4:**
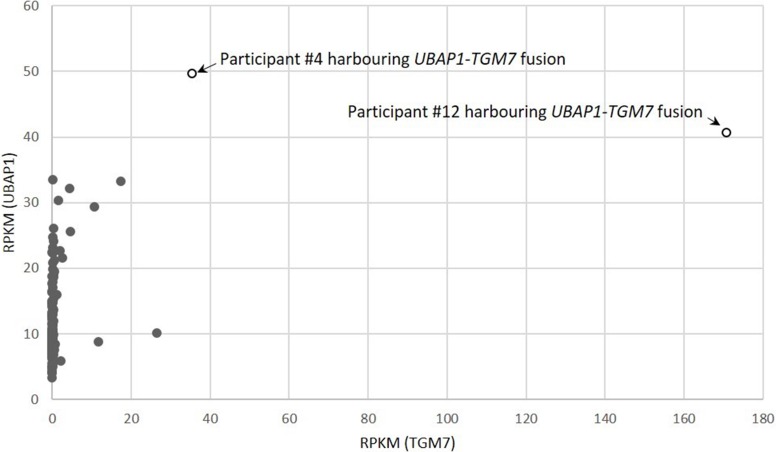
Gene expression correlation between fusion genes partners *UBAP1* and *TGM7* in 220 EOC tumors studied Tumors harboring the *UBAP-TGM7* fusion are indicated. Gene expression is measured as Reads Per Kilobase of transcript per Million (RPKM) mapped reads.

We set out to investigate what role recurrent fusion genes may play in the tumorigenesis of EOC, particularly the rarer EOC histological types. We identified one recurrent fusion (*UBAP1-TGM7*) unique to CC tumors. *UBAP1-TGM7* was present in 2 of 20 (10%) CC tumors, making it relatively common in this histological type. To our knowledge, this fusion gene has not previously been reported in EOC, or any other tumor type [12]. It was also not been observed in over 200 samples spanning 27 different non-neoplastic human tissues [23]. The *UBAP1-TGM7* fusion identified here is predicted to fuse the first exon of *UBAP1* (coding for 75 amino acids) to the 10^th^ exon of *TGM7*, preserving the gene partners reading frames. As shown in Figure 4, our RNA-seq data indicates that *TGM7* expression is dramatically increased in tumors harboring the *UBAP1-TGM7* fusion (17-fold and 87-fold increase above average). *UBAP1* is a component of the endosomal sorting complex required for transport I (ESCRT-I), a complex that functions in the sorting of ubiquitinated cargo [24, 25]. Residues in the N-terminus of Ubap1 are responsible for interacting with other proteins in the ESCORT-I complex [24], and are retained in the fusion. *TGM7* codes for a transglutaminase, and functions to stabilize protein assemblies through the formation of gamma-glutamyl-epsilon lysine crosslinks. The portion of Tgm7 retained in the *UBAP1-TGM7* fusion contains the glutaminase domain, and conceivably this function is preserved. Neither *UBAP1* nor *TGM7* has a function that, based on known functionally relevant cancer-specific fusions (typically kinases, transcription factors, chromatin modifiers [1, 26]), is readily connected to tumorigenesis, therefore, it is difficult to speculate what role this fusion might serve. *UBAP1* has also been reported fused, in-frame, with *UBAP2* in one breast cancer tumor, with *ADAMTSL1* in one low grade glioma [12]. *TGM7* has not been found partnered in-frame with any other genes.

Beyond this CC-specific fusion, no other fusions were present in more than 3% of the samples analyzed, including histology-specific and histologically combined analyses. The next most recurrent fusion was *CRHR1-KANSL1*, detected in 6 tumors (2.7% of all samples). Despite its lower frequency, *CRHR1-KANSL1* is of interest as it reside at the locus 17q21.31, a structurally complex genomic region containing a 900-kb inversion polymorphism, multiple copy-number variants [27, 28], and markers that associate with ovarian cancer [29], female fertility [28], and female meiotic recombination [28]. Further, *CRHR1-KANSL1* fusions have been reported in 5% of TCGA's colorectal tumors and 3.5% of TCGA's uterine carcinosarcomas [12]. This fusion identified here (and in TCGA) involved the exchange CRHR1's 5′-UTR to KANSL1's 5′-UTR. Thus, the Kansl1 protein is predicted to be intact and functional, but potentially with altered expression. Kansl1 plays a role in chromatin modification, a functional class previously described for genes in fusions associated with tumorigenesis. Fused to CHRH1's 5′-UTR, altered Kansl1 expression may lead to aberrant histone acetylation and protein expression. *CRHR1* has also been reported fused with *CENPP*, *KIA0100*, and *SPOP*, in one breast, cervical, and uterine tumor, respectively [12]. *KANSL1* has also been reported fused with *ORMDL3* and *LAYN* in one breast tumor and with *UNC45B* in one lung tumor. Finally, we found no evidence for the recurrent fusion genes previously reported in the literature, including *ESRRA-TEX40* [17], *CDKN2D-WDFY2* [16], and *BCAM-AKT2* [15]. This is consistent with other reports [11–13, 30].

We found some evidence that CC tumors harbor more fusions on average than any other EOC histological type, including HGS tumors. This is unexpected as CC EOC tumors are typically described as having fewer somatic genetic alterations than HGS tumors [31, 32], and CC tumors from other organ sites, particularly renal clear cell cancer [12], have revealed few fusions per sample. This pattern was observed in one batch of RNA-seq data (Batch 2, *p* = 0.002); a second batch trended in this direction but was not significant at *p* < 0.05 (Batch 3, *p* = 0.16). Not enough CC tumors were sequenced in Batches 1 and 4 to add to this result. We note that despite analyzing within RNA-seq batch, in Batch 2, CC tumors had more mapped reads than non-CC tumors (*p* = 0.016, *t*-Test assuming unequal variance) (see Supplementary Table 2 for details). This may have affected the number of fusions detected in the other tumors in this batch, but it is not clear why this occurred. In Batch 3 the number of mapped reads between tumors was not significantly different (*p* = 0.9, *t*-Test assuming equal variance). Therefore, we view this CC results with caution, and emphasize the need for replication of this broad finding.

Despite being the largest non-HGS analysis to date, the main limitation of this study was sample size; hence, we focused inference on subtype-specific common recurrent (> 5% frequency). We also note that differences in RNA-seq library preparation methods made it difficult to compare results across batches. Strengths of this study are the inclusion of the rarer subtypes types of EOC, subtype-specific analyses, and the capacity to make direct comparison to those fusion genes detected in all TCGA tumors [12] based on the use of highly similar methods (i.e. applying PRADA). PRADA has been described as a conservative but accurate tool relative to other fusion callers [19]. This means it may fail to detect certain fusions, but those that it does report, validate more often than when using other tools. Thus, we acknowledge that some fusions may have been missed in our analysis. We only had the capacity to validate one fusion, and therefore we chose to validate the CC-specific fusion gene *UBAP1-TGM7*. The confirmed validation of this fusion lends support to PRADA being an accurate bioinformatics tool for calling gene fusions.

Only two of the recurrent fusions reported in our EOC tumors, *CCDC6-ANK3* and *COL14A1-DEPTOR*, were also found in TCGA HGS EOC tumors. Although recurrent, these were found to be relatively rare here (1.4% of HGS tumors) and in TCGA HGS EOCs (0.5–1% of tumors). To conclude, we identify one in-frame fusion at 10% frequency in the CC EOC subtype, but find little evidence for common (> 5% frequency) recurrent fusion genes in EOC overall, or in HGS tumors.

## MATERIALS AND METHODS

### Ethics statement

All cases provided written informed consent for use of their tissues and medical records in research; all protocols were approved by the Mayo Clinic Institutional Review Boar (HSC # 09-003270 and 09-008768).

### Tumor samples

Patients were those receiving surgery for ovarian, fallopian tube, or primary peritoneal cancer at the Mayo Clinic (Rochester, MN). Clinical diagnoses were confirmed by a gynecologic pathologist, who verified tumor histology, grade, and the presence of 70% tumor content prior to RNA extraction from fresh frozen tissue. RNA was extracted from a total of 220 tumors, including 141 HGS, 55 END, 20 CC, and 4 MC (Supplementary Table 2). Six HGS samples were from relapsed tumors, and six other samples were from primary tumors treated with neo-adjuvant chemotherapy (NACT) (5 HGS, 1 CC). All other samples were from treatment-naive primary tumors. RNA-seq data for 7 normal samples sourced at Mayo Clinic from whole ovary (*N* = 5), endometrium (*N* = 1), and omentum (*N* = 1) was also generated (Supplementary Table 3). RNA-seq data for 10 normal whole ovary samples was downloaded from GTEx (http://www.gtexportal.org/home/) (Supplementary Table 3). Normal samples were analyzed using the same pipeline as tumor samples. Results were used to remove fusion genes that are not cancer-specific (see below). RNA-sequencing of tumor and normal samples was performed in four batches (details in Supplementary Table 4).

### Bioinformatics analysis of fusion genes

Pipeline for RnAseq Data Analysis (PRADA) (v1.2) was used to call gene fusions [33]. Combined genome and transcriptome reference files were downloaded from http://bioinformatics.mdanderson.org/Software/PRADA/, including the hg19 assembly, Ensembl GTF Release 64, and dbSNP 135 v37. Fusions events were filtered to those supported by at least two split reads, one ‘perfect-match’ junction spanning read, and low homology between fusion partner genes (retained those with PRADA E-value > 0.001). The 2*junction length (junL) bases parameter was set to 80% of the read length (recommended). Fusion events identified by PRADA in 17 (7 Mayo Clinic, 10 GTEx Portal) normal tissues were removed. A catalog of 11,531 fusion RNAs identified in over 200 RNA sequencing libraries from 27 different non-neoplastic human tissues analyzed using the SoapFuse pipeline was further used to remove fusion genes that are not cancer-specific [22, 23]. To assess robustness of fusion results from PRADA pipeline, we also ran a subset of sample through three other fusion callers (SoapFuse, FusionMap and TopHat Fusion) with results presented in Supplementary Table 1.

### Association of fusion genes with clinical outcome

The relationship between fusion gene status (none versus any) and prognosis (OS and PFS) was evaluated by fitting a Cox proportional hazards regression model (subtypes combined, and for each subtype separately). Post-NACT, relapsed, and normal samples were removed from outcome analyses. Progression-free survival time was defined as time from the date of diagnosis to the date that second-line therapy was initiated for a clinically-actionable tumor recurrence. Tests for association between fusion status and time to event clinical endpoints were assessed with Wald tests. The association between fusion gene status (presence vs absence) and clinical features were assessed via logistic regression models and Wald tests, with adjustment for histology and batch. All association *p*-values reported are unadjusted for multiple-testing.

### Fusion validation

The UBAP1-TGM7 fusion (Supplementary Figure 1) was validated using SuperScript^®^ VILO^™^ cDNA synthesis kit from Thermo Fisher Scientific. cDNA was amplified with TaqGold at 35 cycles and 60 degree annealing temperature for Sanger sequencing using model 3730 × l DNA analyzer. Primers (5′GCTCTCCCTAGGGGCTGTC, 3′TCCCTAAGACCCCCAGACTC) were designed based on sequence in Supplementary Figure 1 with red indicating the fusion site. All validation work was performed in Genome Analysis Core at the Mayo Clinic.

### SUPPLEMENTARY MATERIALS FIGURES AND TABLES






