# Supplementary material for: Characterization of fusion genes in common and rare epithelial ovarian cancer histologic subtypes
Source: Oncotarget. 2017 Apr 1;8(29):46891–9. doi: 10.18632/oncotarget.16781 (PMC5564530; doi:10.18632/oncotarget.16781)
Supplement: Supplementary file 1 [file oncotarget-08-46891-s001.pdf]

Characterization of fusion genes in common and rare epithelial ovarian cancer histologic subtypes

Supplementary Materials

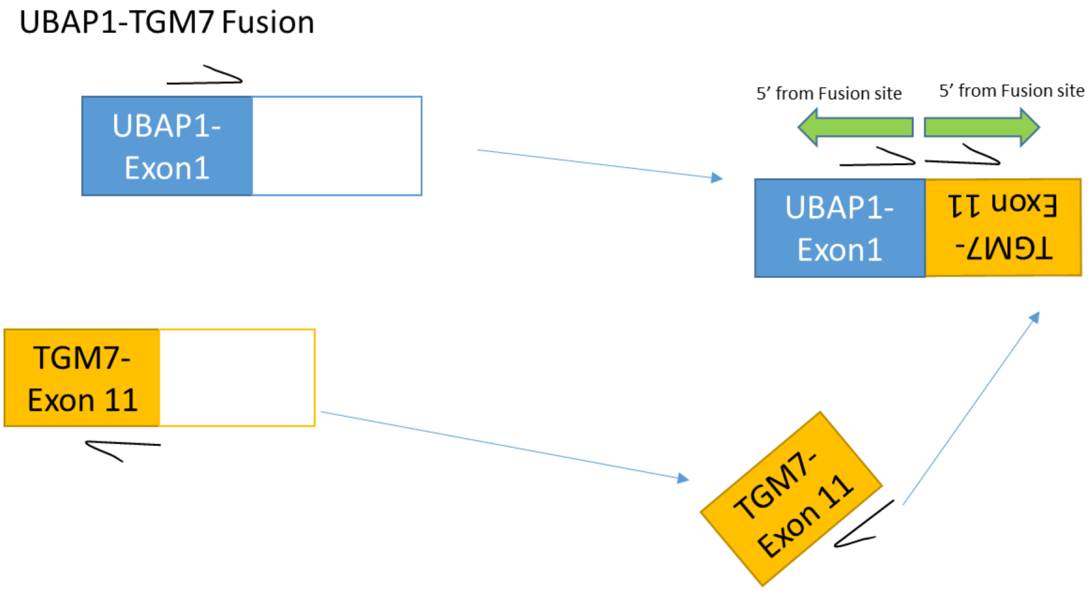

AATAGGACTCGCTGCTAACTCTTTGGGCATACCTTCGCGATTCAAGGCTCTTGGGTTTCCCGCTTTGGCCGCCTCGCCAAACCCAAGTCTTACACTCTTAGTG
ACCCCCTCCTTTCCTCCTTCCCCCTTAGGTCAGCAGGGTTTACTCATTCCAATTGCAATGCTCCATGTTGCCGGAGTAGCTCCTCGCTAGAAGAGAGATTAGTT
ATGTCACCGTCGGCTCCTCCCATTGGTCCGGGCGTGTGGAGCGCGGCGCCATGACGTCAGACGCCCAAATGAGTGGGGCGGTGAGGGGAAGGAGGAGGG
AAGTAGGACTTCAACATGGCGGCTGCGGCACTGGCGGTGGCTACGGTGACGGCCTGGCCCGGAGCGGGCAGAGTTGGAGGTGGTGGCGTTCGCTCTCCC
TAGGGGCTGTCGGGAGCTCAGCGGGGACCGAGCCTGGGAGGCCGGCCGGTGCCAGCACCTTTCGGCTTCTGAGACGGCGGCAGCAGCGGCAT**TCAGGA**
TCCCCTGAGGAGAGAGCTGTCTTCATGAAGGCTTCTCGGAAAATGCTGGGCCCCCAAAGAGCTTCTTTGCCCTT CCTGGATCTCCTGGAGTCTGGGGGTCTT
AGGGATCAGCCAGCGCAGCTGCAGCTTCACCTGGCCAGGATACCCGAGTGGGGCCAGGACCTGCAGCTGCTGCTGCGTATCCAGAGGGTGCCAGACAGCA
CCCACCCTCGGGGGCCCATCGGACTGGTGGTGCGCTTCTGTGCACAGGCCCTGCTGCATGGGGGTGGTACCCAGAAGCCCTTCTGGAGGCACACAGTGCG
GATGAACCTGGACTTTGGGAAGGGTGAGTGTGAGGCAGGCCTGAGAAGGCACCTACAGGGAGGGGAGCTGTGGGTCCATGAACACAGTGGGGTGGGCC
CAGCCAGATTACACACAGACCCCTTCCTCCAACACACACACATTCTGGGCAGTGAAGCTGCCAGCTGCTGCACCTCTGATGCCACCCCACCAGGCGAGA

Supplementary Figure 1: UBAP1-TGM7 fusion location.

Supplementary Table 1: Fusion genes identified in EOC tumor samples and passing filtering steps. See Supplementary_Table_1

Supplementary Table 2: Detailed tumor sample information. See Supplementary_Table_2

Supplementary Table 3: Detailed normal sample information

Participant No.	Source	Organ Site	Batch	No. Fusions Detected	No. Mapped Reads
N1	Mayo Clinic	Ovary	1	0	51907112
N2	Mayo Clinic	Ovary	1	0	18324526
N3	Mayo Clinic	Ovary	1	0	52038734
N4	Mayo Clinic	Ovary	1	1	34403746
N5	Mayo Clinic	Ovary	1	0	34839661
N6	Mayo Clinic	Endometrium	1	0	30223837
N7	Mayo Clinic	Omentum	1	0	28672340
N8	GTEx	Ovary	NA	0	73960262
N9	GTEx	Ovary	NA	0	61642790
N10	GTEx	Ovary	NA	2	62525368
N11	GTEx	Ovary	NA	2	77658463
N12	GTEx	Ovary	NA	7	73018343
N13	GTEx	Ovary	NA	3	72532953
N14	GTEx	Ovary	NA	2	88520755
N15	GTEx	Ovary	NA	0	60244888
N16	GTEx	Ovary	NA	1	77595569
N17	GTEx	Ovary	NA	6	90730304

Supplementary Table 4: RNA-seq batch (1–4) features

	Library Preparation	RNA selection	Sequencing Centre	Read Length	Average Mapped Reads (± s.d)	Number of Tumor Samples	Number of Normal Samples
Batch 1	TruSeq Stranded Total RNA Library Preparation Kit	Total RNA, rRNA depleted	BGI Americas (Beijing, China)	101	45,128,496 ± 12,667,304	95	7
Batch 2	TruSeq Stranded Total RNA Library Preparation Kit	Total RNA, rRNA depleted	Epigenome Core, University of South California (Los Angeles, CA)	50	86,799,336 ± 23,817,596	17	0
Batch 3	TruSeq RNA Library Preparation Kit v2	mRNA	Medical Genome Facility, Mayo Clinic (Rochester, MN)	101	79,484,713 ± 7,226,239	30	0
Batch 4	TruSeq RNA Library Preparation Kit v2	mRNA	Medical Genome Facility, Mayo Clinic (Rochester, MN)	101	100,847,048 ± 18,454,007	78	0
